# Doppler Ultrasound of Vascular Complications After Pediatric Liver Transplantation: Incidence, Time of Detection, and Positive Predictive Value

**DOI:** 10.1055/a-1961-9100

**Published:** 2022-11-16

**Authors:** Martijn V. Verhagen, Ruben H.J. de Kleine, Hubert P.J. van der Doef, Thomas C. Kwee, Robbert J. de Haas

**Affiliations:** 1 Department of Radiology, UMCG, Groningen, Netherlands; 2 Department of Surgery, Division of Hepato-Pancreato-Biliary Surgery and Liver Transplantation, University of Groningen, University Medical Centre Groningen, Groningen, Netherlands; 3 Department of Pediatric Gastroenterology, UMCG, Groningen, Netherlands

**Keywords:** hepatic veins, areas, structures & systems, hepatic arteries, ultrasound-color doppler, methods & techniques, ultrasound-spectral doppler, transplantation, themes

## Abstract

**Purpose** Doppler ultrasound (DUS) is widely used to detect vascular complications after pediatric liver transplantation (LT). This study aimed to assess the moment of first detection of vascular complications with DUS, and to determine the positive predictive value (PPV) of DUS.

**Materials and Methods** Patients aged 0–18 years who underwent LT between 2015 and 2019 were retrospectively included. 92 LTs in 83 patients were included (median age: 3.9 years, interquartile range: 0.7–10.5). Patients underwent perioperative (intra-operative and immediately postoperative) and daily DUS surveillance during the first postoperative week, and at 1, 3, and 12 months. Vascular complications were categorized for the hepatic artery, portal vein, and hepatic veins. DUS findings were compared to surgical or radiological findings during the 1-year follow-up.

**Results** 52 vascular complications were diagnosed by DUS in 35/92 LTs (38%). 15 out of 52 (28.8%) were diagnosed perioperatively, 29/52 (55.8%) were diagnosed on postoperative days 1–7, and 8/52 (15.4%) after day 7. The PPV for all vascular complications diagnosed with DUS was 92.3%. During the 1-year follow-up, 18/19 (94.7%) hepatic artery complications, 19/26 (73.1%) portal vein complications, and 7/7 (100%) hepatic vein complications were diagnosed perioperatively or during the first week.

**Conclusion** The majority of vascular complications during the first year after pediatric LT were diagnosed by DUS perioperatively or during the first week, with a high PPV. Our findings provide important information regarding when to expect different types of vascular complications on DUS, which might improve DUS post-LT surveillance protocols.

## Introduction

Liver transplantation (LT) is the only available treatment for end-stage liver disease in children. Over the last decades, improvements in graft perfusion techniques, surgical procedures, and an increasing proportion of living donor transplantations have led to improved survival rates 1 2 3 4 . Donor organs are scarce, and thus graft survival is of utmost importance. In the last decade in Europe, loss of grafts occurred in up to 7.5% of pediatric LTs, of which the majority happened in the first month 4 . Therefore, optimal surveillance by clinical assessment, laboratory examination, and imaging is essential for early detection and treatment of complications, thereby reducing graft loss 5 6 7 .

Doppler ultrasound (DUS) is the primary bedside imaging technique during and after liver transplantation (LT) in both children and adults. It provides high-resolution dynamic imaging of the liver parenchyma and vascularization, and is inexpensive and radiation-free. Although the diagnostic performance of DUS is operator-dependent, assessment of pediatric LT is generally highly feasible in children due to their lean body mass. Computed tomography (CT) and magnetic resonance imaging are mainly reserved for preoperative assessment of anatomy and liver volume, characterization of focal lesions, and further analysis of postoperative collections and vascular complications in case the DUS findings are inconclusive 8 9 .

The primary aim of DUS is to detect clinically occult vascular complications such as thrombosis, stenosis, and kinking 10 11 12 . In addition, DUS provides information on biliary complications and fluid collections such as hematomas 13 . As mentioned before, early detection of complications followed by subsequent medical, interventional radiological, or surgical treatment aims to improve post-transplant outcome 2 14 15 .

Intraoperative and postoperative DUS for LT is considered the gold standard of care. However, a large temporal heterogeneity of DUS imaging protocols has been reported 16 17 18 19 20 . This heterogeneity might be caused by limited evidence concerning the optimal timing of postoperative DUS and regarding the most efficient postoperative DUS protocol 16 18 20 . For hepatic artery thrombosis (HAT) a large review in children showed a median time to detection of 4.8 days (range: 1.0–9.6) postoperatively 17 . However, for other vascular complications (e. g., portal vein thrombosis), descriptions of incidence are limited to early or late complications, with analysis of incidence in the first 2 weeks often grouped together 10 20 21 . Therefore, it remains unclear when different types of vascular complications may be first diagnosed by DUS.

Although various cut-off values for the diagnosis of vascular complications on DUS are available, test performance of DUS during peri- and postoperative surveillance in children is not clearly established 21 . This is partly because DUS is already an established imaging modality. Therefore, a normal DUS examination will not be confirmed by further imaging or surgery and true and false negatives are not verified. A false-positive DUS result, however, has immediate clinical significance and leads to further imaging (e. g., CT) or surgery. Determining the positive predictive value (PPV) may add to a better understanding of DUS test performance after pediatric LT.

Because of the limited evidence available, the primary objective of this study was to assess the moment of first detection of vascular complications with DUS during standardized peri- and postoperative DUS surveillance after pediatric LT; the secondary objective was to determine the positive predictive value of DUS.

## Materials and methods

### Patients

This single-center retrospective study was performed in our national pediatric liver transplant center. The study was approved by the local research ethics committee and the need for informed consent was waived.

All consecutive patients<18 years old who underwent LT between April 2015 and June 2019 were eligible for the study. Patients were excluded if they died prior to the first DUS. For repeat LT, the primary and secondary LT were included as separate entries during the 1-year follow-up.

Data collection and reporting of analysis were performed according to the Strengthening the Reporting of Observational Studies in Epidemiology (STROBE) guidelines 22 . Clinical data, DUS images and reports, and other types of imaging were collected during the 1-year follow-up from our prospectively maintained institutional database.

Collected demographic data included: age, gender, and primary disease. Disease-related data at the time of listing for LT consisted of cirrhotic or non-cirrhotic liver disease, model for end-stage liver disease score (MELD, all ages), pediatric end-stage liver disease score (PELD,<12 years), international normalized ratio (INR), bilirubin level (umol/L), albumin level (g/L), and creatinine level (umol/L). Collected surgical variables were type of donor procedure (living or deceased donation) and type of liver transplant (full size or partial liver).

### DUS surveillance protocol and reference standard

All patients underwent DUS according to our local standardized protocol. This protocol consists of intraoperative DUS after all vascular and surgical anastomoses are made, immediately after wound closure, and after arrival in the pediatric intensive care unit (day 0), daily on days 1–7, at 1 month, at 3 months, and after 1 year. To improve the readability of the paper, intraoperative and immediate postoperative DUS examinations are sometimes referred to as perioperative DUS. DUS examinations were performed by a team of five dedicated radiologists and one specialized sonographer. However, for each individual LT one person of this dedicated team was assigned 24 hours a day for 7 consecutive days to perform all perioperative and first week DUS examinations.

### DUS criteria and registration of vascular complications

The DUS criteria used in this study for the detection of vascular complications were any vascular thrombosis or anastomotic stenosis as described in pediatric LT literature 6 21 :

- Hepatic artery: The peak systolic velocity (PSV) and RI (PSV minus end diastolic velocity, divided by PSV) were obtained. A hepatic artery resistive index<0.5 combined with a tardus parvus spectral trace (including a systolic acceleration time>80ms), or (if visible) an anastomotic PSV≥200 cm/s, was considered suggestive of a significant stenosis. The segmental hepatic artery branches were also assessed.
- Portal vein: An anastomotic PSV of>125 cm/s or a pre- to anastomotic ratio of≥4 was considered abnormal. If the anastomosis could not be clearly defined, the narrowest caliber of the hilar portal vein was considered the anastomosis. In the case of an interposition graft or patch, the narrowest caliber was sampled. The segmental portal vein branches were also assessed.
- Hepatic vein(s): The hepatic vein waveform was sampled in all veins approximately 2 cm proximal to the anastomosis. Monophasic flow was considered abnormal.

Vascular complications were categorized by vessel type (hepatic artery, portal vein, hepatic vein(s)). In the case of multiple vessel-specific complications during follow-up (e. g., HAT on day 0 and – after revascularization – again on day 5), all complications were registered. In the case of different types of vessel complications at one time point, all were registered.

All ultrasound examinations were performed on a Toshiba Aplio 500 ultrasound machine (Canon, Ōtawara, Japan). Images had been stored and were available from our Picture Archiving and Communication System.

Abnormal DUS findings were directly compared to subsequent diagnostic or therapeutic actions, such as surgical findings (including repeat surgery), radiological angiography, or further imaging by CT during the first year after LT, as the best available reference standard. Based on the confirmation or disproof of the abnormal DUS result, the vascular complication was further categorized (thrombosis, stenosis, kinking - including loss of signal from compression of the abdominal wall on the graft -, extrinsic compression by a fluid collection), and registered as a true positive or false positive. Abnormal DUS findings without subsequent imaging or therapeutic actions (i. e., without a reference standard) were reported but not included in the analyses.

### Biliary abnormalities and fluid collections

Biliary abnormalities were registered at the time of one of the following interventions: biliary leakage or anastomotic stenosis requiring surgery, endoscopic retrograde cholangiography with balloon dilatation or stenting, or percutaneous transhepatic cholangiodrainage. In addition, the time of clinical consensus of ischemic type biliary lesions (ITBL) was registered. For fluid collections (hematoma, loculated ascites, abscess), interventions were either surgery or radiological drainage. Fluid collections detected on DUS without treatment were not included in this study. In the case of repeated biliary or fluid collection interventions during follow-up, all were registered as separate entities.

### Statistical analysis

Data analysis was descriptive. Continuous variables were summarized using median and interquartile range (IQR). Kaplan-Meier curves and bar charts were used to illustrate the time points of vascular complication detection by DUS (GraphPad Prism version 9.0, GraphPad Software, La Jolla California USA), categorized per vessel, during the first 2 weeks, and during the 1-year follow-up. In addition, Kaplan-Meier curves were used to illustrate the moment of DUS vascular complication diagnosis compared to that of biliary and collection-related interventions during the first 2 weeks and at the 1-year follow-up. Last, Kaplan-Meier curves of DUS-detected vascular complications were stratified for age groups (0–2 versus>2 years) and graft type (LDLT, DDLT full size, DDLT split liver), and differences between curves were tested using the Mantel Cox log-rank test.

## Results

### Patient and liver transplantation characteristics

The entire cohort of 93 consecutive LTs in 83 children was included in the study. One patient died without undergoing DUS and was excluded. The median patient age at the time of LT of the remaining 92 LTs was 3.9 years (IQR 0.7–10.5, min.-max. 0.2–16.8). Table 1 further describes the patient and LT characteristics.

**Table:** **Table 1** Patient and liver transplantation characteristics.

Number of LTs	92 (100%)
Age at the time of LT (years), median (IQR)	3.9 (0.7–10.5)
Gender, male, N (%)	53 (57.6%)
Cirrhotic disease, N (%)	76 (82.6%)
MELD score*, median (IQR)	18 (13–23)
PELD score*, median (IQR)	9 (2–18)
Bilirubin (umol/L)*, median (IQR)	118 (36–273)
Period on waiting list, days, median (IQR)	104 (44–174)
INR*, median (IQR)	1.3 (1.1–1.6)
Albumin (g/L)*, median (IQR)	36 (30–40)
Creatinine (umol/L)*, median (IQR)	22 (16–39)
Full size	24 (26.1%)
DDLT	24 (100%)
Split liver	68 (73.9%)
LDLT	33 (48.5%)
DDLT	35 (51.5%)

*at the time of listing for LT, INR: international normalized ratio, IQR: interquartile range, LT: liver transplantation, MELD: model for end-stage liver disease, N: number, PELD: pediatric end-stage liver disease, LDLT: living donor liver transplantation, DDLT: deceased donor liver transplantation.

**Fig. 1:**
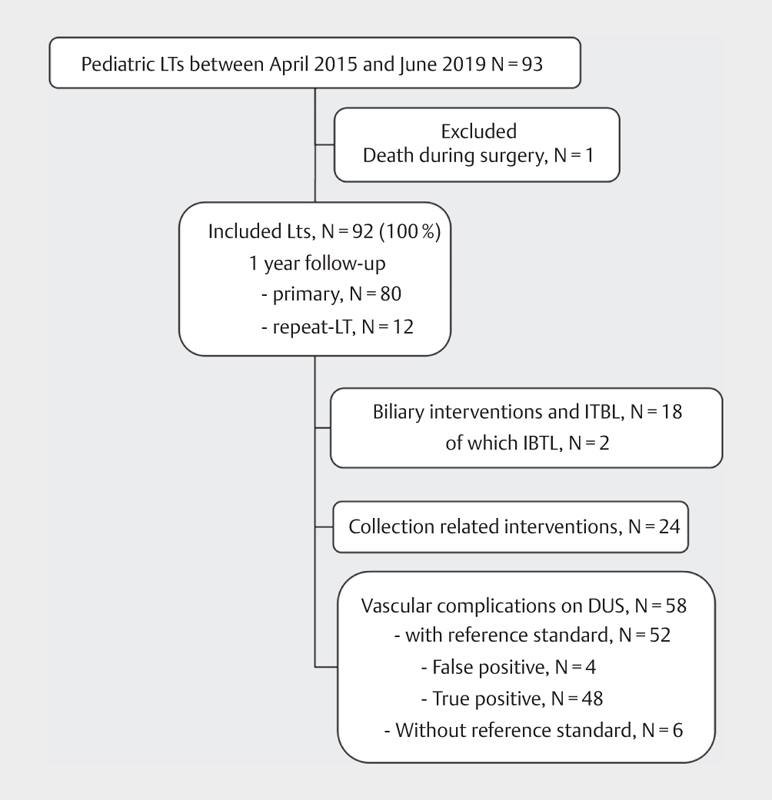
Population flowchart.

Fig. 1 displays the study population flowchart. **Supplemental Table 1** displays all primary diseases and baseline data. **Supplemental Table 2** illustrates the distribution of the anastomotic techniques used.

### Moment of detection of vascular complications on DUS after LT

**Fig. 2:**
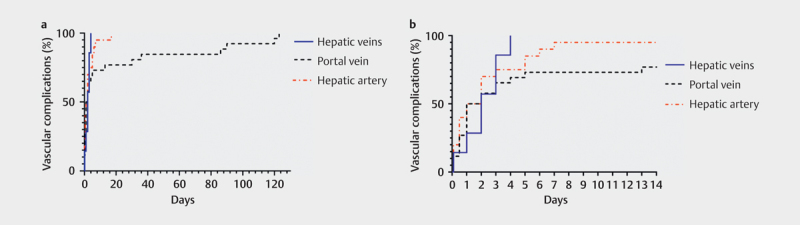
Kaplan-Meier (inverted) curves of vascular complications diagnosed by DUS during 1-year follow-up, detailed for day 0–130 (Left ( **a** )) and day 0–14 (right ( **b** )), categorized for the hepatic artery, portal vein, and hepatic veins.

44 out of 52 (84.6%) vascular complication diagnoses on DUS during the 1-year follow-up were made either perioperatively or in the first week after LT ( Tab. 2 , Figs. 2 3 ). Of these, 15/52 (28.8%) were perioperative: 7/52 (13.5%) intraoperative and 8/52 (15.4%) immediately postoperative. On postoperative days 1–7, 29/52 (55.8%) vascular complications were detected by DUS, while from day 8 to 1 year post-LT, this was the case in 8/52 (15.4%) LTs. **Supplemental Figures 1 and 2** demonstrate Kaplan-Meier curves of vascular complication detection categorized for age groups (0–2 versus>2 years) and graft types (LDLT, DDLT split liver, and DDLT full size), respectively, without significant differences.

**Fig. 3:**
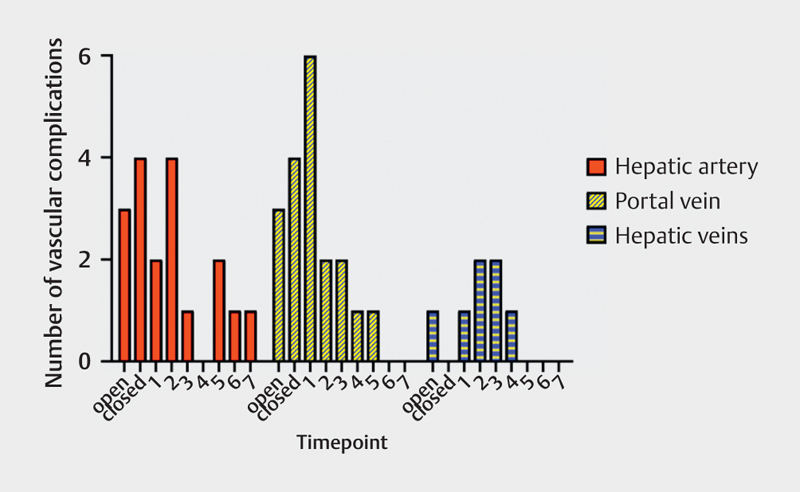
Histogram of vascular complications diagnosed by DUS, perioperatively and during the first postoperative week.

**Table:** **Table 2** Vascular complication diagnosis by DUS categorized per period. For each vessel the reference standard diagnosis and the true- and false-positive cases are specified.

			Perioperative	Days 1–7	Days 8–365	Total
**Hepatic artery**						
	Thrombotic	TP	2	2	1	5
		FP		1		1
	Kinked	TP	4	4		8
	Extrinsic compression	TP		2		2
	Significant stenosis	TP	1	2		3
	Total		7	11	1	19
**Portal vein**						
	Thrombotic	TP	5	5	1	11
	Kinked	TP		2		2
	Significant stenosis	TP		4	6	10
		FP	2	1		3
	Total		7	12	7	26
**Hepatic vein**						
	Thrombotic	TP	1			1
	Extrinsic compression	TP		2		2
	Significant stenosis	TP		4		4
	Total		1	6		7
Total			15	29	8	52

DUS: Doppler ultrasound; TP: true positive; FP: false positive.

19 hepatic artery complications were diagnosed by DUS during the 1-year follow-up. Eighteen of them (94.7%) were diagnosed either perioperatively or in the first postoperative week ( Tab. 2 , Fig. 2, 3 ). True-positive HAT was diagnosed in 5 out of 92 LTs (5.4%), with the median day of diagnosis being 6 days after LT (IQR 0–12 days).

26 portal vein complications were diagnosed during the 1-year follow-up. 19 of them (73.1%) were diagnosed either perioperatively or in the first postoperative week ( Tab. 2 , Fig. 2, 3 ).

7 hepatic vein complications were diagnosed with DUS. All diagnoses were made either perioperatively or in the first postoperative week ( Tab. 2 , Figs. 2, 3 ).

There were 6 instances of an abnormal DUS without subsequent actions (i. e., without a reference standard), which were not included in the analysis. Five out of six patients did not suffer graft loss at the 1-year follow-up. These are further detailed in **Supplemental Table 3** .

### PPV of DUS for vascular complication detection

48 out of 52 vascular complication diagnoses on DUS were true positive, resulting in a PPV of 92.3%.

The PPV for the hepatic artery was 94.7% (18 true positive/19 total). The only false-positive hepatic artery diagnosis was a HAT registered on day 5, which immediately underwent surgery after DUS.

Three false-positive portal vein stenoses were suggested with DUS, 2 postoperatively after wound closure, and 1 at 4 months after LT. CT during follow-up ruled out stenoses in these cases. This resulted in a PPV for the portal vein of 88.5% (23/26).

No false-positive hepatic vein complications were diagnosed on DUS, resulting in a PPV of 100% (7/7).

### Biliary abnormalities and fluid collections

**Fig. 4:**
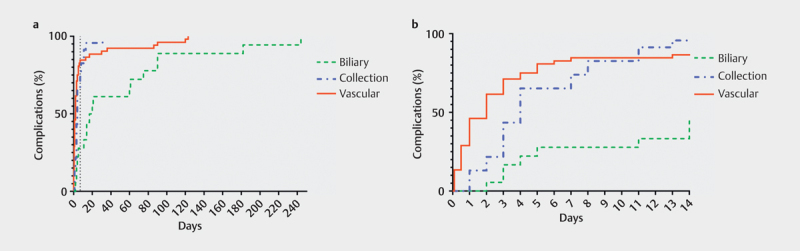
Kaplan-Meier (inverted) curves of vascular complications diagnosed on DUS confirmed by the reference standard, and interventions for biliary abnormalities, and fluid collection interventions during 1-year follow-up. The complications are given for day 0–250 (left) and day 0–14 (right), and categorized for vascular, biliary, and collection-related complications. The vertical dotted line in figure A marks postoperative day 7.

18 biliary complications were registered, including 2 cases of ITBL. 24 fluid collection-related interventions were performed. Fig. 4 illustrates the time points of biliary and fluid collection interventions in comparison to vascular complications diagnosed via DUS.

## Discussion

This study determined at what time points after pediatric LT radiologists can first detect vascular complications with DUS during standardized DUS surveillance. We also determined the PPV of DUS in this setting. We found that the vast majority of vascular complications were diagnosed with DUS perioperatively or during the first postoperative week, with only a small percentage of vascular complications diagnosed in the subsequent one-year follow-up (84.6% versus 15.4%, respectively). The PPV for vascular complication detection by DUS was high (92.3%). As with vascular complications, fluid collections requiring interventions occurred mostly in the first postoperative week, whereas biliary abnormalities were diagnosed mostly at a later time.

DUS surveillance during and after LT is focused on vascular complications, with the aim of detecting these often clinically occult complications as early as possible. The most urgent vascular complication is thrombosis. Early HAT in children carries a hazard ratio for graft loss of up to 14 23 , and appropriate treatment will improve long-term graft and patient survival 14 15 17 23 . Similarly, pediatric portal vein thrombosis is associated with higher mortality, and prompt detection and treatment are instrumental in reducing mortality and graft loss 24 . In our study, the majority of cases of vascular thrombosis in the first year occurred perioperatively (N=8 out of 17) or in the first week (N=7 out of 17). Daily DUS in the first postoperative week in our center allows for a timely diagnosis and treatment of thrombosis, and based on the aforementioned studies, we believe this will likely improve outcomes.

In addition to daily postoperative DUS in the first week, we also perform intraoperative and immediate postoperative DUS as part of our standardized DUS surveillance protocol. This has also been suggested in previous pediatric studies 25 26 , but to our knowledge, its implementation varies between LT centers. Perioperative DUS detected several thromboses and kinked vessels (including loss of signal from compression of the abdominal wall at the graft), which required immediate intervention. The detection rate was similar for intraoperative and immediate postoperative DUS (N=12 and N=13, respectively), and this underlines the importance of performing DUS at both time points.

Peri- and postoperative hemodynamic changes, soft tissue swelling, a hypercoagulable state, and hematomas may also affect the LT 15 27 , and these factors may cause early vascular complications. However, these factors may also lead to a false-positive stenosis diagnosis on DUS, which may be the explanation for the 3 cases of false-positive portal vein stenosis in our study. In the case of perioperative or first postoperative week portal vein stenoses diagnosed by DUS, we suggest DUS follow-up instead of immediate surgical correction.

The main limitation of our study is the suboptimal reference standard caused by the retrospective study design. Although surgery or radiological imaging and interventions are the best option for comparing DUS findings, they are not fully DUS-independent because DUS already has an established role in vascular complication detection. In addition, we could not assess false-negative or true-negative rates for DUS because LTs with normal DUS findings did not undergo further imaging. Last, we found 6 abnormal DUS cases in our cohort without a reference standard. The optimal design for a prospective study would be to verify each DUS finding with CT or surgery. However, this would not be ethically justifiable. To further investigate the usefulness of DUS surveillance after pediatric LT, a prospective observational study with predefined criteria could be of value.

Although our data suggests that perioperative DUS and daily first week postoperative DUS would benefit all pediatric LTs, our methodology was not suitable to investigate whether this surveillance protocol reduced co-morbidity and improved graft survival. Nevertheless, despite this limitation, we believe that our data justifies the use of this protocol at our center. In the absence of studies with superior methodology, our data could provide guidance for other LT centers.

In conclusion, the vast majority of vascular complications during the first year after pediatric LT are diagnosed by DUS during the operation, immediately after the operation, or during the first postoperative week during daily DUS surveillance, with a high PPV. Fluid collection-related complications requiring intervention occur mostly in the first postoperative week, while most biliary abnormalities are diagnosed later. Our findings provide important information for pediatric LT centers regarding when to expect different types of vascular complications on DUS during and after pediatric LT, which might further improve the DUS post-LT surveillance protocol.

## Notice

This article was changed according to the following Erratum on April 12th 2023.

## Erratum

In the above-mentioned article a specification in Table 2 was not correct. The corrected Table 2 is shown below. This was corrected in the online version on April 12th, 2023.
