# Correction: Doppler Ultrasound of Vascular Complications After Pediatric Liver Transplantation: Incidence, Time of Detection, and Positive Predictive Value

**DOI:** 10.1055/a-2061-8073

**Published:** 2023-04-14

**Authors:** Martijn V. Verhagen, Ruben H.J. de Kleine, Hubert P.J. van der Doef, Thomas C. Kwee, Robbert J. de Haas

**Affiliations:** 1 Department of Radiology, UMCG, Groningen, Netherlands; 2 Department of Surgery, Division of Hepato-Pancreato-Biliary Surgery and Liver Transplantation, University of Groningen, University Medical Centre Groningen, Groningen, Netherlands; 3 Department of Pediatric Gastroenterology, UMCG, Groningen, Netherlands

## CORRECTION

Doppler Ultrasound of Vascular Complications After Pediatric Liver Transplantation: Incidence, Time of Detection, and Positive Predictive Value

Martijn Vincent Verhagen, Ruben H.J. de Kleine, Hubert P.J. van der Doef et al.

Ultrasound International Open 2022; 8: 36–42

10.1055/a-1961-9100

published online: 2022-11-16

In the above-mentioned article a specification in Table 2 was not correct. The corrected Table 2 is shown below. This was corrected in the online version on April 14th, 2023.

**Table:** **Table 2** Vascular complication diagnosis by DUS categorized per period. For each vessel the reference standard diagnosis and the true- and false-positive cases are specified.

			Perioperative	Days 1–7	Days 8–365	Total
**Hepatic artery**						
	Thrombotic	TP	2	2	1	5
		FP		1		1
	Kinked	TP	4	4		8
	Extrinsic compression	TP		2		2
	Significant stenosis	TP	1	2		3
	Total		7	11	1	19
**Portal vein**						
	Thrombotic	TP	5	5	1	11
	Kinked	TP		2		2
	Significant stenosis	TP		4	6	10
		FP	2	1		3
	Total		7	12	7	26
**Hepatic vein**						
	Thrombotic	TP	1			1
	Extrinsic compression	TP		2		2
	Significant stenosis	TP		4		4
	Total		1	6		7
Total			15	29	8	52

DUS: Doppler ultrasound; TP: true positive; FP: false positive.

