# Supplementary material for: Doppler Ultrasound of Vascular Complications After Pediatric Liver Transplantation: Incidence, Time of Detection, and Positive Predictive Value
Source: Ultrasound Int Open. 2022 Nov 16;8(2):E36–42. doi: 10.1055/a-1961-9100 (PMC9668490; doi:10.1055/a-1961-9100)
Supplement: Supplementary file 1 — Supplementary Material [file 10-1055-a-1961-9100-0249.pdf]

Supplementary material

Supplemental Table 1: Primary diseases and additional baseline data of the study population

		Age (years) at LT	PELD (<12 y) *	MELD (all ages) *	Bilirubin level (umol/L) *	INR*
Primary disease	Number	Mean (SD)	Mean (SD)	Mean (SD)	Mean (SD)	Mean (SD)
**Cirrhotic liver disease**						
Biliary atresia	39	2.8 (4.2)	7.1 (10.0)	16.2 (5.5)	148.6 (118.4)	1.3 (0.6)
Alagille syndrome	8	6.6 (5.3)	2.3 (7.2)	16.3 (3.9)	180.1 (107.9)	1.2 (0.1)
Autoimmune hepatitis	2	12.9 (1.7)	12 (0)	15 (5.7)	32 (5.7)	1.8 (0.7)
Primary sclerosing cholangitis	3	14.3 (3.6)	12	15 (7)	143.7 (198.1)	1.3 (0.2)
Progressive familial intrahepatic cholestasis	4	10.7 (7.0)	-11 (9.9)	13 (4.5)	98.5 (128.3)	1.3 (0.3)
Alfa1-antitrypsin deficiency	5	6.4 (3.1)	8.6 (12.4)	11.9 (19.4)	126.8 (209.1)	2.1 (1.0)
Wilson's disease	6	12.8 (2.7)	15 (2.8)	35.6 (6.9)	385 (301.8)	3.1 (10.0)
Cystic fibrosis	2	17.7 (0.1)	28 (0)	29.5 (10.6)	229.5 (306.2)	1.8 (0.3)
Gestational autoimmune liver disease	1	0.3	41	40	444	5.2
Nephronophthisis type 3	1	5.2	0	10	35	1.1
Neonatal sclerosing cholangitis	1	12.5	N/A	11	24	1.3
Neonatal cholestatic of unknown origin	3	3.8 (5.2)	6.3 (10.5)	17 (5)	210.7 (139.0)	1.2 (0.3)
Cirrhosis, unknown cause	1	8.4	-12	7	11	1.1
**Non-cirrhotic liver disease**						
Acute fulminant hepatitis	9	4.9 (4.2)	26.3 (16.3)	32.9 (10.8)	303.4 (198.3)	4.3 (2.2)
Primary hyperoxaluria	3	7.5 (2.6)	28.3 (21.9)	24.3 (4.0)	20.5 (15.3)	1.3 (0.5)
Hepatoblastoma	2	3.4 (2.6)	0	0	4.5 (0.7)	1 (0.1)
Methylmalonic academia	1	3.9	30.0	30.0	2.0	1.6
Graft-versus-host disease	1	10.5	9	20	406	1.1
**Total**	92	5.9 (5.6)	11.2 (14.6)	20 (9.6)	170.1 (169.0)	1.8 (1.3)

*at the time of listing for LT; LT: liver transplantation; INR: international normalized ratio; MELD: model for end-stage liver disease; N: number; N/A: not applicable; PELD: pediatric end-stage liver disease score; SD: standard deviation

Supplemental Table 2: Anastomotic technique

	Number (%)
Number of LTs	92 (100%)
Biliary anastomosis	
Duct-duct	24 (26.1%)
Roux-en-Y hepaticojejunostomy	68 (73.9%)
Portal vein anastomosis	
End-end with interposition graft	22 (23.9%)
End-end	70 (76.1%)
Hepatic artery anastomosis	
End-end	88 (95.6%)
End-side	1 (1.1%)
End-end with interposition graft	3 (3.3 %)
Hepatic vein anastomosis	
End-side piggy bag	90 (97.8%)
End-end classic	2 (2.2%)

LT: liver transplantation

Supplemental Table 3: Vascular complications on DUS without reference standard

**Gender and age**	**Primary disease, transplant type**	**Vascular complication diagnosed by DUS**
Boy, 10 years old	PSC<br>Full size, heart-beating	Portal vein: acceleration of pre-anastomotic flow velocity of 27cm/s to 161 cm/s at the anastomosis (ratio 5.9) on postoperative day 6. Followed with DUS and normalized after 5 months. No graft loss at 1-year follow-up.
Boy, 11 years old	Wilson's disease<br>Split liver, heart-beating	Portal vein: acceleration of pre-anastomotic flow velocity of 30cm/s to 289 cm/s at the anastomosis (ratio 9.6) on postoperative day 3. Graft failure with high-grade hepatic encephalopathy necessitating high urgency repeat LT without further assessment of the portal vein.
Boy, 12 years old	Autoimmune hepatitis<br>Split liver, heart-beating	Portal vein: thrombosis in the segment 4 branch of a segment 2-4 split-liver, in combination with parenchymal necrosis during transplant. Accepted with no further need for imaging or interventions. No graft loss at 1-year follow-up.
Girl, 1 year old	Alagille<br>Split liver, living	Portal vein: acceleration of pre-anastomotic flow velocity of 50 cm/s to 157 cm/s at the anastomosis (ratio 3.1) after wound closure. Followed with DUS and normalized at 1 month. No graft loss at 1-year follow-up.
Boy, 4 years old	Alagille<br>Full size, heart-beating	Hepatic artery: intrahepatic RI of 0.35 with tardus parvus waveform, but normal extrahepatic RI, on day 6. Followed with DUS and normalized at 3 months. No graft loss at 1-year follow-up.
Boy, 6 years old	Alagille<br>Full size, non-heart-beating	Hepatic artery: intrahepatic RI 0.43 with tardus parvus waveform intraoperatively. Normalized at the PICU, but seen again on day 6, after which normalization occurred and no more abnormality was seen on DUS. No graft loss at 1-year follow-up.

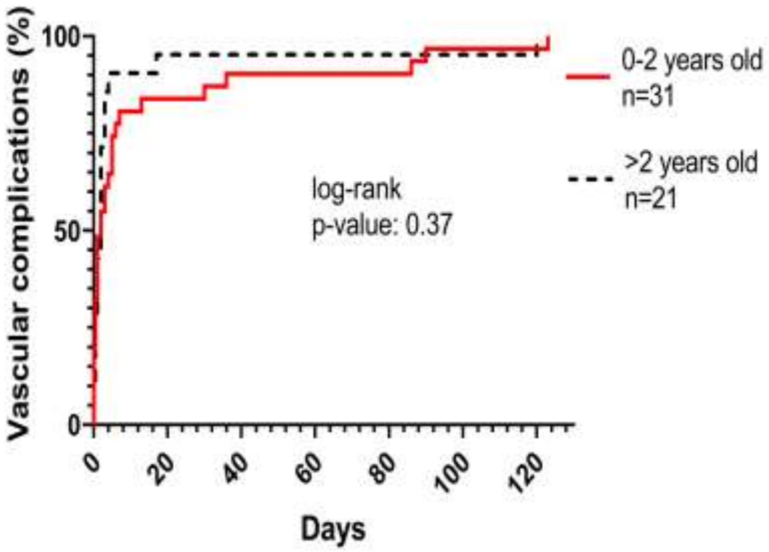

Supplemental Figure 1: Kaplan-Meier (inverted) curves of vascular complications diagnosed by DUS during 1-year follow-up, categorized for age groups 0-2 years old and >2 years old.

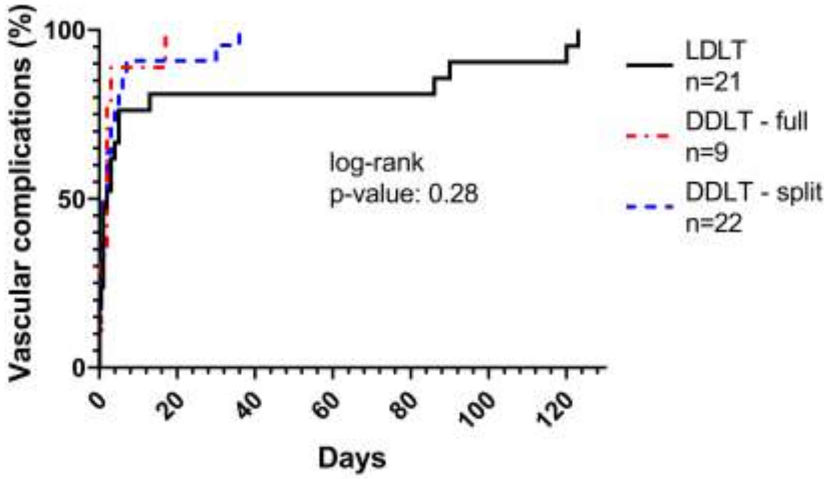

Supplemental Figure 2: Kaplan-Meier (inverted) curves of vascular complications diagnosed by DUS during 1-year follow-up, categorized for graft type. LDLT: living donor liver transplantation; DDLT: deceased donor liver transplantation
